# Role of psychiatric associations in improving public attitudes towards mentally ill

**DOI:** 10.1192/j.eurpsy.2026.10401

**Published:** 2026-07-31

**Authors:** K. Başar

**Affiliations:** Department of Psychiatry, Hacettepe University, Ankara, Türkiye

## Abstract

**Abstract:**

Stigma on mental health struggles and psychiatric practices are important components of difficulty in accessing mental healthcare, in addition to their contribution to the burden experienced with health challenges. These kinds of stigma are rooted in a wide range of factors including structural inequities and centuries old myths. Still, some elements of the mainstream discourse and some practices of the mental healthcare practitioners can play reproducing and enhancing roles in the stigmatizing attitudes. Stigmatizing attitudes about the professional identity, role, and practices may also lead to difficulties in the professional life of the practitioners. Therefore, the widespread presentation of the professional identity of a psychiatrist and clinical psychiatric practices may be influential. Professional organizations are often considered the representatives of these facets of the profession, especially in the messages they share with the community. They can act as reliable sources of information for both the lay community, other healthcare professionals, and the administrators. The associations have to work on the means they can reach to wider audience in their messages, but also in the way their discourse could modify the common stigmatizing attitude. Often information alone is not sufficient to change the general attitude, the association has to be open on the critic of the practices that have been associated with violation of rights, and the management of the members of the profession who fail to stick to the core features of the professional identity and ethical principles. For all these work, it is essential that a kind of collaboration could be constituted with people with lived experience. This kind of collaborations not only increase the impact of the work, but also work as models of nonstigmatizing behavior.

**Disclosure of Interest:**

None Declared

